# Therapeutic Effect of a Recombinant Human Fibronectin Construct in Skeletal Muscle Repair and Oxidative Stress

**DOI:** 10.3390/ijms262110700

**Published:** 2025-11-03

**Authors:** Yuqi Chen, Yuxuan Fan, Yichao Dong, Xiaoqin Yu, Jianen Gao, Xu Ma

**Affiliations:** National Research Institute for Family Planning, Chinese Academy of Medical Sciences & Peking Union Medical College, Beijing 100081, China; chenqiberg@163.com (Y.C.);

**Keywords:** fibronectin, aging, skeletal muscle, microenvironment, injury repair

## Abstract

Aging mice experience a depletion of muscle extracellular matrix fibronectin (FN). Therefore, enhancing FN expression in the aging tissue microenvironment may be able to maintain satellite cell function and facilitate the repair of damaged skeletal muscle. Herein, we have used molecular dynamics (MD) simulations to select FN functional domains, which were combined into a single construct, rhFN-NM (recombinant human Fibronectin-N-terminal module). The antioxidant properties of this construct were tested at the cellular level and included effects on cell adhesion, anti-aging, apoptosis and expression of aging-related proteins. When used in an animal skeletal muscle injury model, naturally aging mice, or in *IL-10^(^*^−/−)^ gene knockout mice, this construct promoted skeletal muscle repair and improved the immune microenvironment of the tissue. Overall, we show that the construct rhFN-NM improves skeletal muscle repair and protects against oxidative stress.

## 1. Introduction

Aging is a complex process that affects the function of various tissues, leading to a health decline and an increased susceptibility to disease [1], with typical manifestations that include losses of muscle fiber quality, strength, endurance and metabolic capacity [2]. Sarcopenia is the age-related progressive loss of muscle mass and strength that can lead to fractures [3], with a mechanism and pathophysiology that have not been fully elucidated [4].

Muscle stem cells (MuSCs) are located at the periphery of muscle fibers, between the sarcolemma and the basal lamina, and are also known as Satellite Cells [5]. MuSCs are crucial for skeletal muscle regeneration [6]. After an injury, they exit their quiescent state and proliferate into myogenic progenitor cells, thus generating new muscle fibers and replacing damaged tissue [7,8]. In young individuals, MuSCs are abundant and able to rapidly respond to injuries and promote muscle recovery. In the elderly, however, the MuSC population may be up to 30% lower. This is concurrent with a decline in muscle function and repair capacity and an increased risk of sarcopenia. The reduction in MuSCs numbers in the elderly muscle is associated with what is known as “inflammaging”, a chronic inflammatory state where stress pathways are activated, e.g., the focal adhesion kinase(FAK) and p38 mitogen-activated protein kinase (MAPK) [9]. This activation may suppress MuSC proliferation and differentiation capability, possibly due to a change in the local microenvironment [10].

Fibronectin (FN) is an extracellular glycoprotein that can bind to muscle cells [11] via, for example, laminin and integrin-α7β1, the primary integrin in skeletal muscle [12]. An increase in integrin-α7β1 can enhance the migration of MuSCs to the site of injury [13]. MuSCs can secrete FN when activated, which is crucial for muscle stem cell function and maintenance and for immune regulation and repair of skeletal muscle injury. Therefore, FN supplementation can in principle be used to treat muscle injury. However, due to its large molecular weight, the expression of full-length recombinant FN is challenging. Instead, FN is usually extracted from plasma or after segmental recombinant expression, but these methods are resource-intensive and carry safety risks.

The optimal functional domain for recombinant expression can be selected using molecular dynamics simulations (MDs). Herein, we have identified a domain in FN that binds integrin-α7β1. We show that an recombinant human fibronectin N-terminal module(rhFN-NM) construct can enhance the adhesion and vitality of aging cells and can also regulate the microenvironment of skeletal muscle in aging mice, facilitating the repair and regeneration of damaged tissues.

## 2. Results

### 2.1. Integrin and Fibronectin Structures and Analysis of Binding Modes

**Figure 1:**
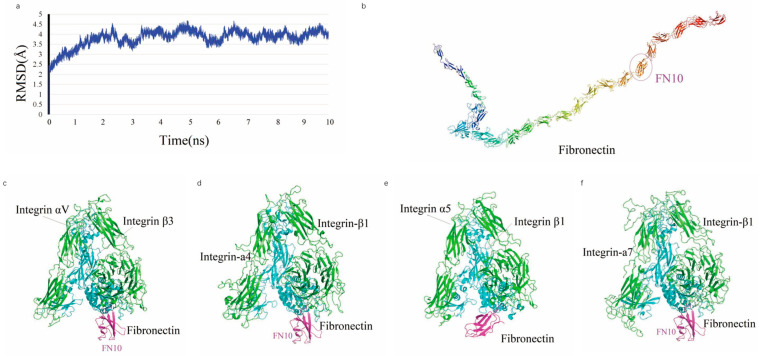
MD profiles and FN-integrin interactions. (**a**) temporal evolution of RMSD values (*y*-axis, in Å) for full-length FN backbone atoms over a 10 ns simulation (*x*-axis, in ns); (**b**) structural representation of full-length FN, highlighting its domain organization; (**c**–**f**) modeled structures of the FN10 domain on FN in complex with various integrin heterodimers: (**c**) αVβ3, (**d**) α4β1, (**e**) α5β1, and (**f**) α7β1, illustrating the binding conformations.

In an MDs simulation, full-length FN achieved stability after 4 ns, as shown by the temporal variation in the Root Mean Square Deviation (RMSD) (Figure 1a), indicating that the system reached an equilibrium state with an average RMSD of 4.03, 2.76, 2.78 and 3.57 Å, respectively. The average structure of FN is characterized by a V-shaped extended chain comprising 25 tandem β-sheet repeats (FN1–FN25) (Figure 1b), with the FN6 module positioned at the junction between two linear segments formed by FN1–FN5 and FN7–FN25. The crystal structure of the integrin ectodomain αV/β3 heterodimer in complex with FN10 (PDB: 4MMX) (Figure 1c), along with complexes involving other ectodomains (Figure 1d–f), exhibits a high degree of conservation at the interaction interfaces.

**Figure 2:**
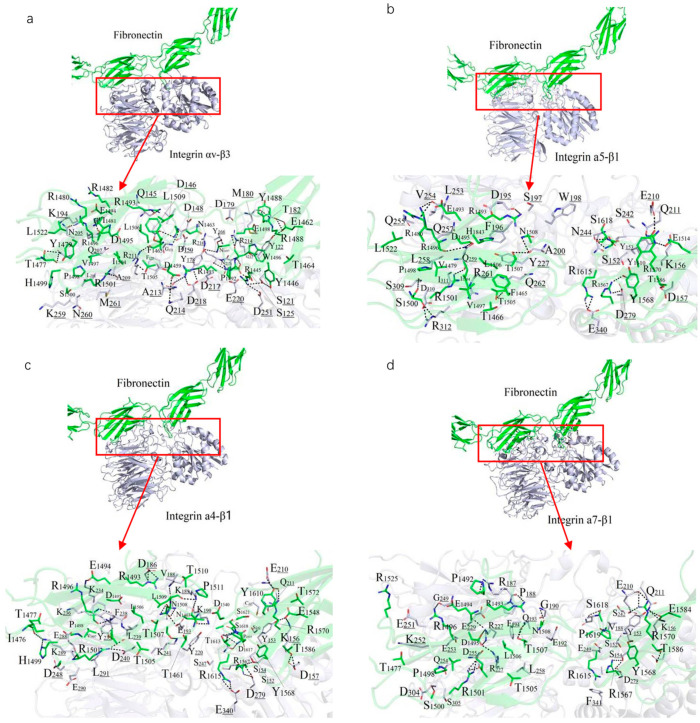
Predicted binding mode between FN and integrin isoforms. (**a**–**d**) potential binding interface between FN (green) and αVβ3 (**a**), α4β1 (**b**), α5β1 (**c**) and α7β1 (**d**), colored in purple. residues participating in the interaction (sticks) and hydrogen bonds (dashes) marked as black are indicated on the right.

A 10 ns MDs was performed to refine the binding models of FN with four integrin subtypes (α4-β1, α5-β1, αv-β3, and α7-β1). The binding interfaces primarily involve the FN10 region, with specific interaction regions spanning residues 1459–1624 across the four complexes (Figure 2). The binding regions for each integrin subtype are as follows: α7-β1 (residues 1477–1620), αv-β3 (1459–1621), α4-β1 (1461–1624), and α5-β1 (1465–1620).

Polar interactions, particularly salt bridges and hydrogen bonds, dominate the stabilization of these complexes through nonbonded electrostatic interactions. A robust polar interaction network was observed at the binding interfaces, with FN residue R1501 forming critical hydrogen bonds with all four integrin subtypes. Common FN regions (R1493–N1508, R1567–R1570, V1582–T1586, and Y1610–S1621) contribute to both hydrophilic and hydrophobic interactions across all complexes. Specifically, the α7-β1/FN complex involves 29 residues from integrin and 39 from FN, forming an extensive interaction network.

Hydrophobic interactions also play a significant role in stabilizing the complexes. Notably, the benzene ring of F1465 and L1509 from all integrin subtypes forms hydrophobic contacts with V188 of FN, enhancing binding stability (Figure 2). The binding energies, calculated as −111.33 kcal/mol (α4-β1), −102.34 kcal/mol (α5-β1), −102.09 kcal/mol (αv-β3), and −95.27 kcal/mol (α7-β1), reflect the varying strengths of these interactions, with α4-β1 exhibiting the strongest binding, likely due to its extended binding region and enhanced polar interactions. Overall, the interplay of strong salt-bridge-mediated polar interactions and hydrophobic contacts at the binding interface is pivotal to the stability of FN-integrin complexes.

### 2.2. Recombinant Fibronectin Construction and Integrin Binding Interactions

**Figure 3:**
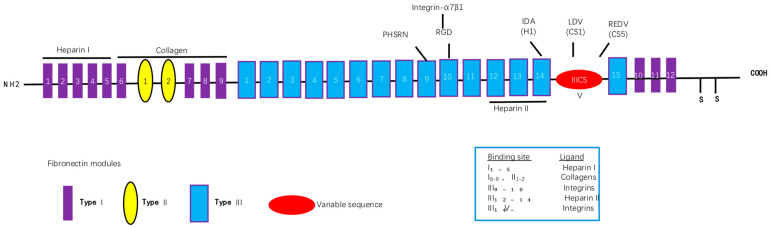
Simplified fibronectin structure.

**Figure 4:**
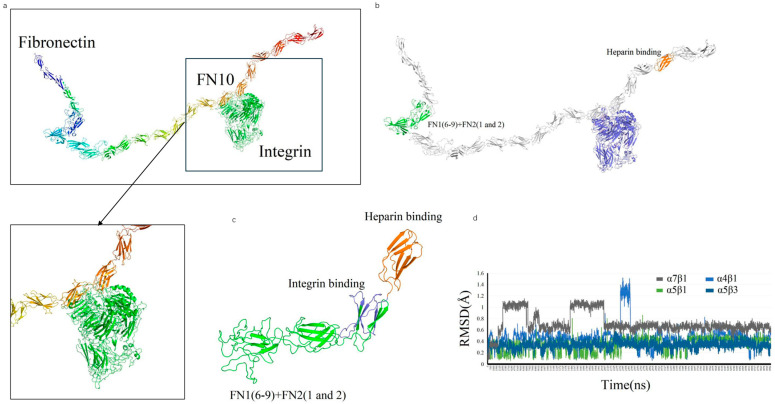
De novo modeling of rhFN-NM. (**a**) structure of fibronectin; (**b**) recombined FN protein; (**c**) 3D diagram of modeling rhFN-NM. The structure of FN1(6–9) + FN2(1 and 2) and heparin region are green and orange, respectively; (**d**) RMSD values (*y*-axis) versus time (*x*-axis) for four integrin subtypes (αVβ3, α4β1, α5β1, α7β1) with rhFN-NM.

We designed a novel recombinant protein (rhFN-NM) by fusing the collagen-binding domain (FN1(6–9) + FN2(1–2)) and heparin-binding domain of fibronectin (FN) (Figure 3). Structural alignment confirmed that the local conformations of these functional domains in rhFN-NM remained nearly identical to their native counterparts in full-length FN (RMSD < 1.0 Å). However, the overall architecture of rhFN-NM exhibited significant divergence from native FN, primarily due to the engineered integrin-binding linker region. This linker adopted a novel spatial arrangement, altering the relative positioning between the collagen- and heparin-binding domains (Figure 4). To investigate the functional implications of these structural changes, molecular dynamics simulations were performed, revealing distinct interaction patterns between rhFN-NM and integrin receptors. The dominant binding modes extracted from trajectory clusters (see Section 4) were further analyzed to elucidate the mechanistic basis of the modified interactions.

The RMSD change curves of four Integrin subtypes (αVβ3, α4β1, α5β1, α7β1) with the recombinant Fibronectin-derived protein rhFN-NM complex. The calculated maximum RMSD fluctuation value for each complex was under 1.6 Å, all reaching equilibrium after 5 ns (Figure 4d)—a demonstration of structural stability in the binding systems—with each average structure further used for subsequent analysis.

**Figure 5:**
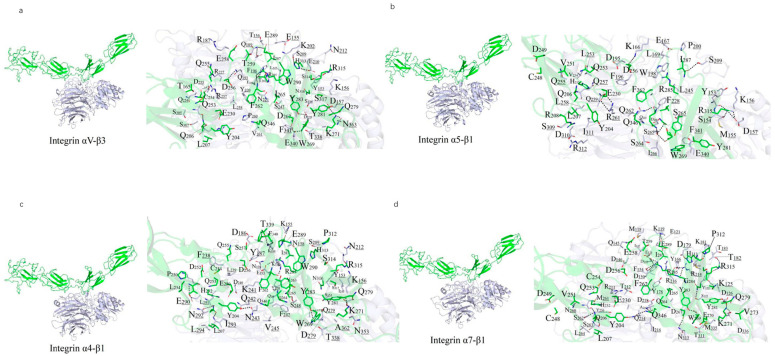
Binding mode prediction between different integrin isoforms and rhFN-NM. (**a**–**d**). potential binding interface between rhFN-NM (green) and αVβ3 (**a**), α5β1 (**b**), α4β1 (**c**) and α7β1 (**d**) (purple). residues participating in the binding interface (sticks) and hydrogen bonds (dashes) are indicated (right panels).

rhFN-NM showed weaker binding to the four integrins αVβ3, α4β1, α5β1 and α7β1 (−85.29, −89.77, −82.14 and −98.06 kcal/mol, respectively) than full length FN. For the αVβ3/rhFN-NM complex, 35 and 45 residues participated in the interaction (Figure 5a), consistent with the low binding energy observed. The lowest binding affinity was for the complex involving α5β1, with 23 and 37 residues from the integrin and rhFN-NM, respectively (Figure 5b). For αVβ3 and α4β1 complexes, 30 and 37 residues, respectively, participated with 39 residues on rhFN-NM (Figure 5a,c). Thus, it could be inferred that rhFN-NM protein binds with different integrin isoforms with a similar binding region (Y204-Q345).

### 2.3. rhFN-NM Protein Mitigates Oxidative Stress-Induced Cellular Damage

Oxidative stress causes cell dysfunction, DNA damage and ultimately, irreversible injury and death due to an imbalance between oxidant and antioxidant mechanisms. FN can affect cell proliferation, differentiation and vitality through various mechanisms. These include remodeling of the extracellular matrix, enhanced inflammation, and alterations in mitochondrial function.

**Figure 6:**
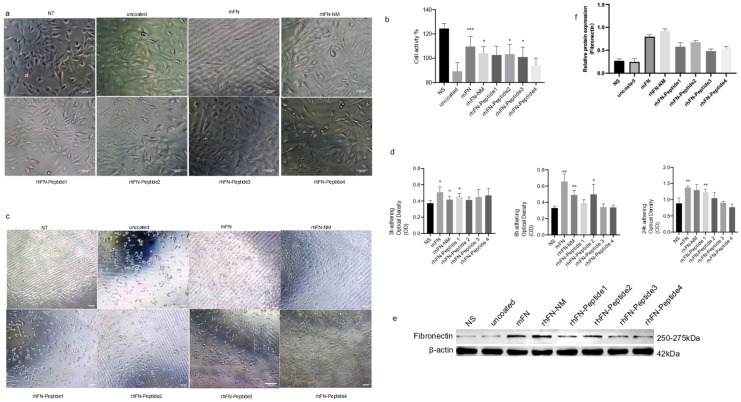
(**a**) Effect of proteins and peptides on the proliferation of C2C12 myoblasts (scale bar 100 μm); (**b**) the histogram represents the percentage of cell viability after injury in each group; (**c**) cell morphology and cell viability after oxidative stress injury; (**d**) cell adhesion; (**e**,**f**) effect of rhFN-NM protein and rhFN-Peptide on FN expression. coated groups: rhFN-NM, mFN, rhFN-Peptide1, rhFN-Peptide2, rhFN-Peptide3, rhFN-Peptide4; Untreated group: NT; data are presented as mean ± SEM (*n* = 5 biological replicates per group) * *p* < 0.05; ** *p* < 0.01; *** *p* < 0.001.

We tested the effect of rhFN-NM, mFN (Native Mouse Fibronectin, Abcam) and other peptides on the proliferation and anti-aging capability of C2C12 cells. To evaluate anti-aging effects, plates were pre-coated with the different constructs, seeding 10^5^ cells, and culturing them for 24 h. Oxidative stress damage was induced with 400 µM H_2_O_2_. Morphological changes and viability were assessed using the CCK-8 assay. After 24 h of culture on the pre-coated plates, no significant differences were observed with respect to the control in the treatment groups, except in the rhFN-Peptide4 group, where a slightly lower cell count was observed (Figure 6a). Following oxidative stress treatment, all groups experienced morphological changes. The rhFN peptide groups (rhFN-Peptide1–4) showed varying degrees of cell shrinkage and detachment, whereas the rhFN-NM and mFN groups were the least affected (Figure 6b). The cell viability in the latter two groups was also confirmed by CCK-8 results (Figure 6d).

To further demonstrate the effect of FN on cell function, we used the CCK-8 assay to measure the OD values of adherent cells after 3, 6 and 24 h after seeding (Figure 6d), which measured the adhesion of the constructs tested. After 3 h, strong adhesion was evident for rhFN-NM, mFN protein, and rhFN-Peptide1, with a significant difference in adhesion observed at 24 h. In contrast, poor adhesion was observed for rhFN-Peptide3 and rhFN-Peptide4. The expression levels of FN in the treated cells were elevated for all constructs, especially for rhFN-NM and mFN protein (Figure 6e). This suggests that the treatments may have an impact on the extracellular matrix and the observed protective effects against oxidative stress.

In summary, the results indicate that rhFN-NM and mFN may have beneficial effects on C2C12 cells, promoting better tolerance to oxidative stress and potentially enhancing their anti-aging potential compared to the peptide controls. The increased expression of Fibronectin in these treated groups suggests that changes in the extracellular matrix may be involved in the observed protective effects. Further research is necessary to fully understand the mechanisms underlying these observations.

### 2.4. rhFN-NM Protein Inhibits Cellular Senescence and Apoptosis

To detect and stain senescent cells, we used the β-galactosidase staining method, based on the upregulation of senescence-associated β-Gal (SA-β-Gal) activity. After oxidative stress damage to the pre-coated cells, the cells were fixed and stained for β-galactosidase activity, followed by blue cell colonies counting under the microscope to assess the aging status of each group. rhFN-NM, mFN, rhFN-Peptide1, and rhFN-Peptide2 groups had a lower positive rate.

**Figure 7:**
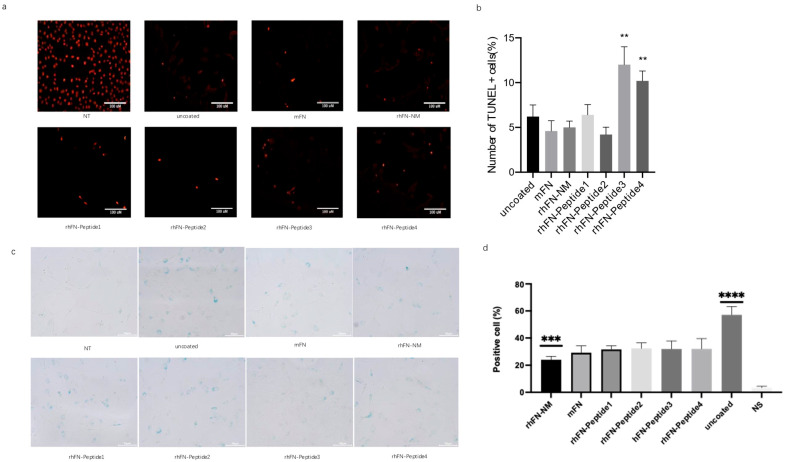
(**a**,**b**) Cell apoptosis detection by TUNEL labeling. (**c**,**d**) β-galactosidase staining for cell senescence. note: coated groups: rhFN-NM, mFN, rhFN-Peptide1, rhFN-Peptide2, rhFN-Peptide3, rhFN-Peptide4. uncoated group: uncoated; pos is the positive control treated with DnaseI, data are presented as mean ± SEM (*n* = 5 biological replicates per group) ** *p* < 0.01; *** *p* < 0.001; **** *p* < 0.0001. scale bar 100 μm.

To confirm the inhibitory effect of the constructs on apoptosis, apoptosis rates were measured using the TUNEL assay. Positive rates of apoptotic cells were lower in the rhFN-NM, mFN, rhFN-Peptide1 and rhFN-Peptide2 groups than in the uncoated control group (Figure 7).

### 2.5. rhFN-NM: Regulating Mouse Skeletal Muscle Microenvironment

The skeletal muscle microenvironment consists of extracellular matrix (ECM), cytokines, growth factors and immune cells, among others. These components regulate the muscle stem cell (MuSC) behavior essential for muscle regeneration and repair.

We measured the levels of inflammatory cytokines (IL-1β, IL-6, TNF-α and IL-10) in the serum samples from Geriatric mice following CTX muscle injury using ELISA(Mouse IL-6 (H007), Mouse IL-10 (H009-1), Mouse IL-18 (H002), and Mouse TNF-α (H052-1);Nanjing Jiancheng Bioengineering Institute; Nanjing, China). In the rhFN-NM group, reduction was observed for pro-inflammatory IL-6 and TNF-α, but anti-inflammatory IL-10 increased. In the mFN group, the levels of pro-inflammatory cytokines, such as IL-6, TNF-α and IL-1β were reduced, while the concentration of the anti-inflammatory cytokine IL-10 increased (Table 1). However, both groups showed similar patterns of change. Treatment with rhFN-NM or mFN produced an increase in IL-10 levels that was not significant (45% higher and 61% higher than control, respectively), suggesting that enhanced anti-inflammatory response may partially contribute to repair, albeit requiring further validation.

**Figure 8:**
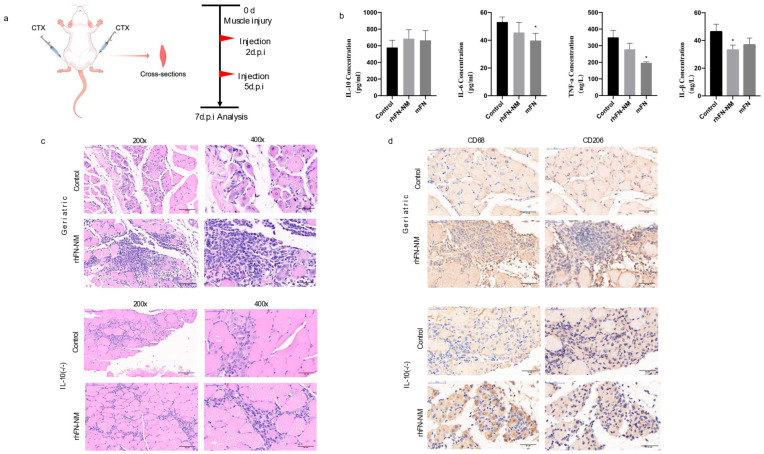
(**a**) Treatment scheme in aging mice: after CTX-induced muscle injury, geriatric mice received saline (control), rhFN-NM, or mFN injections at 2 and 5 days post-injury (d.p.i.), with samples collected at 7 d.p.i.; (**b**) effect of rhFN-NM on inflammatory cytokines; (**c**) representative immunohistochemical images of muscle in control and rhFN-NM-treated mice (200×/400×); (**d**) Immunostaining of muscle macrophages (CD68, CD206) in control and rhFN-NM-treated mice. data are presented as mean ± SEM (*n* = 4–6 mice per group) * *p* < 0.05 (400×).

The H&E staining of skeletal muscle tissue sections from two groups of mice was analyzed for comparison (Figure 8). The rhFN-NM subset within the Geriatric group displayed marked infiltration of inflammatory cells at the injury site, accompanied by a significant aggregation of immune cells. Notably, the injury area of the skeletal muscle featured numerous centrally nucleated muscle fibers, a hallmark of muscle fiber regeneration. In the Control group, however, there was only a slight infiltration of inflammatory cells, with a few centrally nucleated muscle fibers observed at the injury site, indicating modest muscle fiber regeneration.

Different types of macrophages play distinct roles in muscle injury repair. M1 macrophages secrete inflammatory factors that inhibit repair, whereas M2 macrophages secrete anti-inflammatory factors that facilitate repair. In the aging state, especially in damaged skeletal muscle, macrophages tend to switch from the M2 to the M1 type. We used macrophage marker CD68 and M2 macrophage marker CD206 for immunohistochemical labeling of skeletal muscle tissue. After skeletal muscle injury, the rhFN-NM group of geriatric mice exhibited significant inflammatory cell infiltration, which was confirmed as macrophages by CD68 labeling. Additionally, a large number of macrophages were labeled as CD206-positive in consecutive sections at the same site, indicating a substantial presence of M2 macrophages at the injury site. These macrophages would produce anti-inflammatory factors that promote repair. The control group showed only minimal cell infiltration, with fewer CD206-labeled cells, and no significant presence of M2 macrophages.

### 2.6. rhFN-NM Promotes Skeletal Muscle Regeneration

**Figure 9:**
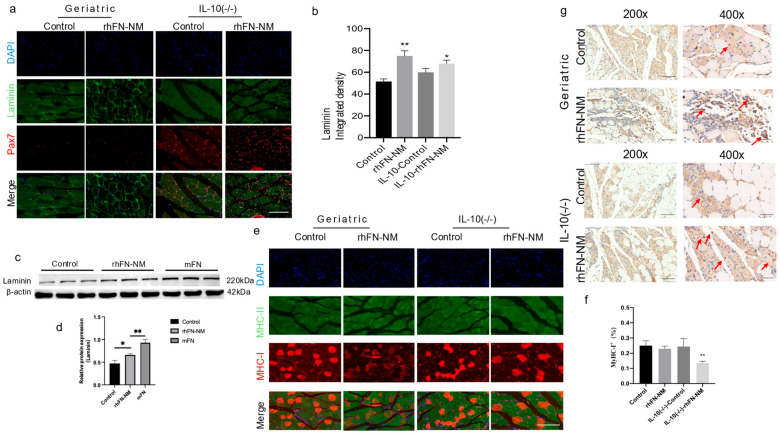
rhFN-NM promotes muscle regeneration and modulates fiber type composition. (**a**) immunofluorescence staining of skeletal muscle in geriatric and *IL-10^(−/−)^* mice after CTX-induced injury, showing Pax7 (red), Laminin (green), and DAPI (blue). groups: control (saline), rhFN-NM, IL-10-Control (saline in *IL-10*^(−/−)^), and IL-10-rhFN-NM; (**b**) quantified fluorescence intensity (scale bar: 200 μm); (**c**,**d**) western blot of Laminin expression in aging mice: control (saline), rhFN-NM, and mFN (positive control). data are presented as mean ± SEM (*n* = 4–6 mice per group) * *p* < 0.05; ** *p* < 0.01. (**e**) muscle fiber type analysis: immunofluorescence of MyHC-I (red) and MyHC-II (green) in geriatric and *IL-10*^(−/−)^ mice. control (saline) vs. rhFN-NM groups; (**f**) western blot of MyHC-I expression in aging mice: control (saline), rhFN-NM, and mFN (positive control). statistical significance: * *p* < 0.05, ** *p* < 0.01; (**g**) rhFN-NM enhances MYH3 embryonic myosin expression, red arrows mark MYH3-positive cells/fibers. (200×/400×).

In the context of aging skeletal muscle, expression of laminin and FN is crucial for the microenvironment and repair of skeletal muscle. We used immunofluorescence double staining to examine the expression of laminin and Pax7 in mouse skeletal muscle (Figure 9). As shown in representative images (Figure 9a), the Pax7 positivity rate in the rhFN-NM group was higher than in the Control group, whereas in the IL-10-rhFN-NM group it was higher than in the IL-10-Control group. Similarly, in the rhFN-NM group, the average fluorescence intensity of laminin was higher than in the Control group, whereas, in the IL-10-rhFN-NM group, it was higher than in the IL-10-Control group (Figure 9b). We further confirmed these findings at the protein level by Western blot analysis for FN and laminin. In the skeletal muscle of aging mice, laminin expression decreased, but both laminin and FN showed increased expression after treatment with rhFN-NM protein and mFN protein (Figure 9c).

Furthermore, we fluorescently labeled slow (type I) and fast (type II) muscle fibers and statistically analyzed their composition. The statistical results indicated a reduction in the proportion of slow type I fibers in the geriatric group and the *IL-10*^(−/−)^ group compared to the control. This proportion was restored following treatment with rhFN-NM protein (Figure 9d). Concurrently, expression of the embryonic myosin heavy chain (MYH3), a marker of regeneration, was enhanced near the muscle injury site in geriatric and *IL-10*^(−/−)^ mice treated with rhFN-NM, suggesting active muscle regeneration (Figure 9g, arrows).

In summary, these results suggest that both rhFN-NM and mFN have potential benefits in promoting muscle repair and regeneration, potentially by mitigating inflammation and fostering an anti-inflammatory microenvironment.

## 3. Discussion

Our study demonstrates that the engineered recombinant FN derivative rhFN-NM effectively enhances skeletal muscle repair in aging mice by mitigating oxidative stress, promoting satellite cell activation and modulating the immune microenvironment. By integrating MD-guided domain selection with functional validation, we developed a novel construct that bypasses the challenges of full-length FN expression while retaining critical integrin-binding and matrix-remodeling capabilities. These findings align with prior studies emphasizing the role of FN in maintaining muscle stem cell niche integrity [1], yet extend the field by introducing a safer, non-plasma-derived alternative with translational potential. In vitro, rhFN-NM significantly improved C2C12 myoblast adhesion and resilience to oxidative damage, while in vivo administration in geriatric mice accelerated muscle regeneration, as evidenced by elevated Pax7+ satellite cells, laminin expression, and M2 macrophage polarization. Notably, rhFN-NM exhibited comparable efficacy in *IL-10^(−/−)^* models, suggesting integrin-dependent mechanisms independent of IL-10-mediated anti-inflammatory pathways.

The lack of significant IL-10 elevation in rhFN-NM-treated groups may reflect microenvironment complexity in aged muscle, where multiple cytokines regulate inflammation resolution, rather than IL-10 alone. Although this study focused on a core set of inflammatory mediators (IL-1β, IL-6, TNF-α, IL-10), we acknowledge that a broader cytokine profiling, encompassing key regulators such as IL-4, TGF-β, and IFN-γ, would provide a more comprehensive understanding of immune modulation. These cytokines play central roles in orchestrating distinct macrophage phenotypes and tissue outcomes during muscle regeneration: IL-4 and IL-13 drive pro-regenerative M2 macrophage polarization [14]; TGF-β is a master driver of fibrosis, yet also has complex dual roles in regulating regeneration [15,16]; IFN-γ is associated with the pro-inflammatory M1 phenotype and the timely resolution of inflammation [17,18,19]. A comprehensive assessment of these mediators would significantly deepen our understanding of the immunomodulatory potential of rhFN-NM.

Furthermore, we recognize that muscle regeneration is a multi-week process and that the promising histological improvements observed at the seven-day time point—a key phase for early regenerative events as established in prior work [20]—require validation through extended functional assessments. Future studies will incorporate longer time points (e.g., 14, 21 and 28 days) and direct functional measurements, such as in vivo muscle contractile force testing, to definitively correlate the structural benefits of rhFN-NM with meaningful functional recovery. Correlating histological repair with functional outcome measures is considered the gold standard for assessing efficacy in skeletal muscle regeneration research [21].

As a novel recombinant protein therapeutic, comprehensive preclinical safety and immunogenicity profiling are essential prerequisites for the clinical translation of rhFN-NM. Although this proof-of-concept study primarily focused on establishing preliminary efficacy and mechanism of action, we have planned follow-up studies using relevant animal models to systematically evaluate its toxicity, biodistribution, and potential anti-drug antibody responses to ensure clinical safety.

However, this study has limitations that warrant consideration. First, the promising histological improvements observed—such as reduced fibrotic area and increased myofiber cross-sectional area—while providing a strong structural basis for expecting functional benefit [22,23], were not coupled with direct functional measurements such as in vivo contractile force or endurance tests. Correlating structural repair with functional recovery is the gold standard in muscle regeneration research, and we acknowledge that the definitive functional efficacy of rhFN-NM remains to be established in future studies. Second, while controlled laboratory conditions ensured experimental rigor, key physiological variables influencing aging populations—such as nutritional status and physical activity levels—were not standardized. Age-related malnutrition (e.g., protein deficiency) may exacerbate FN depletion and alter therapeutic responses [24,25], and sedentary laboratory conditions might underestimate the benefits of rhFN-NM in active individuals, where exercise can synergistically enhance satellite cell function [26,27]. Subsequent studies should incorporate dietary interventions (e.g., low-protein diets) and exercise regimens to evaluate the robustness of rhFN-NM in clinically relevant scenarios.

Regarding methodological rigor, we have clearly specified the sample size (*n*) in each corresponding figure legend and detailed the rationale for sample size determination in the Section 4, which was based on preliminary data and common practices in the field. We commit to performing formal a priori power analysis in future confirmatory studies to ensure sufficient statistical power and reproducibility.

From a clinical translation perspective, rhFN-NM offers distinct advantages over existing therapies. Compared to plasma-derived FN, it eliminates batch-to-batch variability and pathogen risks [28] while maintaining comparable efficacy in promoting laminin deposition and M2 macrophage recruitment. Compared to stem cell therapies, which face implantation challenges and complex delivery requirements [29,30], rhFN-NM acts as a microenvironment modulator suitable for local injection, offering a significant practical advantage for focal injuries. However, challenges remain for broader applications, particularly in treating diffuse sarcopenia. Developing systemic delivery strategies (e.g., nanoparticle encapsulation [31]) and combination therapies with exercise or nutritional supplements (e.g., leucine [32]) may further enhance its efficacy.

To address these challenges, we have formulated a systematic preclinical development roadmap, including (i) comprehensive safety assessment—conducting 90-day toxicity studies in non-human primates to evaluate immunogenicity and organ-specific effects [33]; (ii) delivery system optimization—developing thermosensitive hydrogels for sustained drug release at injury sites [34]; (iii) humanized model validation—utilizing muscle organoids derived from aged donors under variable nutrient/stress conditions for functional verification [28]; and (iv) in-depth mechanistic exploration—identifying novel extracellular matrix-integrin interaction targets enhanced by rhFN-NM via single-cell RNA sequencing. This roadmap directly addresses the core issues raised by the reviewer regarding long-term functional evaluation, comprehensive immune profiling, and safety validation.

By implementing this research plan, rhFN-NM has the potential to evolve into a versatile therapeutic platform, applicable not only to age-related muscle atrophy but also extendable to trauma rehabilitation and degenerative myopathies, ultimately bridging the gap from basic research to clinical application.

## 4. Materials and Methods

### 4.1. Construction of Integrin and Fibronectin

Structures of integrin α-chains (α4/α5) and β-chains (β3) were obtained from the PDB database (PDB ID: 4IRZ/4MMX and 4MMX) [35,36,37,38,39]. Structures of α7 and β1 were obtained by homology modeling using template 4MMX with sequence identity of 28.44% and 45.26%, respectively (Table 2).

The structure of the long sequence of FN was modeled using de novo and multiple-template approaches by means of homology modeling [40,41,42,43,44]. Six regions (residues 48–140, 93–182, 183–275, 298–603, 604–803, 1173–1539 and 1814–2081) were retrieved from PDB structures 109a, 2cg7, 1fbr, 3m7p, 2ha1, 3tlw and 4gh7 (Table 3). Three crystal structures (3tlw, 1fnf and 4gh7) were selected as homology modeling templates for five FN regions (783–996, 903–1268, 1270–1626, 1542–1887 and 1632–1898) with sequence identity of 27.6, 99.7, 82.1, 33.3, and 31.84%, respectively. No templates were found for regions 1–47, 276–297 and 2081–2446; therefore, we used de novo modeling (Table 2). Template-based and de novo FN modeling regions were joined using USC Chimera (Resource for Biocomputing, Visualization, and Informatics, University of California, San Francisco, CA, USA) [44,45,46]. Residues corresponding to the same amino acid sites in overlapping regions were superimposed, followed by the assembly of backbone heavy atoms (C-C(=O)-NH-C) at the junction points, thereby yielding a full-length protein structure.

### 4.2. Structure Optimization of FN Modeling

MD simulations (10 ns) were performed to eliminate steric clashes. The protein was position-restrained with a force constant k of 100 Kcal/mol^−1^ Å^−2^. Simulations were carried out using AMBER software (version 16) [47], using the AMBER ff99sb force field for the complex. Hydrogen atoms were added to the initial models using the leap module, setting ionizable residues at their default protonation states at neutral pH. The protein was solvated in a cubic periodic box of explicit TIP3P water that extended a minimum distance of 10 Å from the box surface to any atom of the solute. The particle mesh Ewald (PME) [48] method for simulation of periodic boundaries was used to estimate the long-range electrostatic interactions, with a cutoff of 10 Å. All bond lengths were constrained using the SHAKE algorithm. Integration time step was set to 2 fs using the Verlet leapfrog algorithm [49,50]. To eliminate possible clashes between solute and solvent, the entire system was minimized in two steps. First, the protein was restrained with a harmonic potential (k Δx^2^) with k = 100 Kcal/mol^−1^ Å^−2^. Water molecules and counter ions were optimized using the steepest descent method of 2500 steps, followed by the conjugate gradient method for 2500 steps. Second, the entire system was optimized using the first step method without any constraints. These two minimization steps were followed by an annealing simulation, with a weak restraint (k = 100 Kcal/mol^−1^ Å^−2^) for the complex, and the entire system was heated gradually in the NVT ensemble from 0 to 298 K over 500 ps. After the heating phase, a 10 ns MD simulation was performed under 1 atm. A constant temperature of 298 K was selected in the NPT ensemble. The constant temperature was maintained using the Langevin thermostat with a collision frequency of 2 ps^−1^. The constant pressure was maintained using an isotropic position scaling algorithm with a relaxation time of 2 ps.

The FN structure stability during the MD simulation was derived from its RMSD from the initial structure [51]. The RMSD value of all heavy atoms in the entire MD simulation trajectory is shown in Figure 1a. The FN structures were stable after 3 ns (RMSD of 4 Å). From the MDs trajectory, 3000 snapshots were extracted from the last 3 ns to obtain the final FN average structure [40].

### 4.3. Construction and Optimization of the FN/Integrin Complex

Sequence blasting indicated that one crystal structure is available for integrin αVβ3 ectodomain bound to FN10 (ID: 4MMX) [35]. To obtain the structural complexes of full length FN with the four integrin ectodomains, the target ectodomain was superimposed on the crystal structure with UCSF Chimera software version 1.16 (Resource for Biocomputing, Visualization, and Informatics, University of California, San Francisco, CA, USA) [44]. Due to the large size of FN, only domain Fn10 was used. The heavy atoms were not restrained, and 3000 snapshots were extracted from the last 3 ns to obtain the final average structure of each complex.

### 4.4. De Novo Modeling of FN-Derived Constructs

We employed the Rosetta protocol [52] to predict the structures of the FN-derived constructs. The intermediate region (T298 to D353), not associated with any known protein structure, was flanked by sequences at both ends that have known crystal structures. Two additional regions (C1-T197 and S354–S435) have crystal structures and were therefore used for multiple-template modeling.

### 4.5. Molecular Docking of Integrin Ectodomains to FN-Derived Constructs

To explore the possible binding interface between FN-derived construct and the four ectodomains of integrin, we used docking simulations performed with Rosetta software, version 3.13 (The Rosetta Commons, University of Washington, Seattle, WA, USA) [53]. In precise docking, the integrin ligands were randomly placed within ~10 Å from the binding regions of receptor FN. Before the start of every simulation, the ligand was perturbed by 3 Å translations and 8° rotations. During precise docking, the side chains at the binding pocket were not allowed to move. A docking pose with the lowest binding energy from a maximum number of 100 conformations was selected. MD simulations (10 ns) were conducted on the selected pose for energy minimization.

### 4.6. Mice

All animal procedures were approved by the Local Animal Ethics Committee. Female C57BL/6N mice (specific pathogen-free [SPF]) were stratified into three groups: (1) young wild-type (WT) controls (3–4 months; genetically unmodified C57BL/6N background); (2) aged group (26–27-month-old retired breeder mice—defined as mice withdrawn from reproduction programs at 6–7 months and maintained as a validated aging model) and (3) IL-10 knockout (KO) group (5-month-old IL-10^−/−^ mice; C57BL/6N background), used to assess inflammation-accelerated aging phenotypes due to IL-10 deficiency exacerbating chronic inflammation. Mice were obtained from Beijing Weitong Lihua (WT/aged) or Saiye Biotech (IL-10 KO; Guangzhou, China), housed with ad libitum access to food/water. Tibialis anterior muscle injury was induced by injection of 50 μL of 10 μM cardiotoxin (Sigma (St. Louis, MO, USA)) in saline. At 2 and 5 days post-injury (d.p.i.), left hind limbs received 0.5 mg/mL rhFN-NM or mFN (Biopur (Reinach, Switzerland)) while right limbs received saline as internal controls. Muscles were harvested at 7 d.p.i. for analysis. A sample size of *n* = 4–6 mice per group was used for in vivo experiments, determined from preliminary data and consistent with established standards in the field for similar muscle regeneration models.

### 4.7. Cells

C2C12 myoblasts (ATCC^®^ CRL-1772™) were cultured in Dulbecco’s Modified Eagle Medium (DMEM) (Thermo Fisher Scientific, Waltham, MA, USA).supplemented with 10% fetal bovine serum (FBS; Fisher Scientific (Waltham, MA, USA), #11531831) and 1% penicillin-streptomycin (Sigma-Aldrich (St. Louis, MO, USA), #P4333) at 37 °C under 5% CO_2_. Four FN-derived polypeptides (rhFN-Peptide1–4; Sangon Biotech (Shanghai, China)) targeting the integrin-α7β1 binding domain (FN residues 1477–1620) were synthesized. Experimental groups included: Coated substrates: rhFN-NM, native mouse FN (mFN; Abcam (Cambridge, UK), #ab92784), or rhFN-Peptide1–4; Controls: Uncoated wells and untreated cells. Cellular senescence was induced by 1 h exposure to 400 μM H_2_O_2_ in complete medium, followed by 4 h recovery in fresh medium prior to assays. All in vitro experiments were performed with *n* = 5 replicates per condition, as determined by preliminary optimization experiments.

### 4.8. CCK-8 Assay

Cell proliferation and viability were assessed using the Cell Counting Kit-8 (CCK-8, Abmole (Houston, TX, USA)). C2C12 cells were seeded at 10^4^ cells/well in pre-coated 96-well plates and cultured for 3, 6 or 24 h. Subsequently, 10 μL CCK-8 reagent was added to each well, followed by incubation at 37 °C for 4 h. Optical density (OD) was measured at 450 nm. Cell viability was calculated as:

Cell viability (%) = [OD (treatment group) − OD (blank)]/[OD (untreated group) − OD (blank)] × 100

where treatment group corresponds to wells containing cells treated with test materials (rhFN-NM, mFN, rhFN-Peptide1–4) or H_2_O_2_-induced senescent cells, plus CCK-8 solution. Untreated group represents wells with untreated cells (no coating/test material) plus CCK-8 solution. Blank is wells containing no cells, just culture medium and CCK-8 solution.

### 4.9. β-Galactosidase Staining

β-Galactosidase staining [52] was performed with a senescence-associated β-galactosidase staining kit (Solarbio (Beijing, China)). C2C12 cells were placed in 6-well plates and incubated for 48 h. Cells were then washed with PBS and fixed in 4% paraformaldehyde (PFA) for 15 min. Cells were incubated with staining mixture for 16 h at 37 °C. The percentage of positive cells was calculated by counting at least 300 cells in six microscopic fields. Image J software, version 1.53t (National Institutes of Health, Bethesda, MD, USA) was used to count the number of cells.

### 4.10. TUNEL Assays

Cell death was detected using the In Situ Cell Death Detection kit TMR red, Roche Diagnostics GmbH, Mannheim, Germany). Cells were fixed with 4% PFA for 1 h, permeabilized with 0.1% Triton X-100 in PBS and incubated in TUNEL staining solution at 37 °C for 1 h. Fluorescence images were obtained using an Olympus IX83 microscope was manufactured by Olympus Corporation (Tokyo, Japan). 

### 4.11. Histology Staining

Mouse skeletal muscle samples were fixed in 4% paraformaldehyde (PFA) solution at 4 °C overnight, dehydrated through a gradient ethanol series, embedded in paraffin, and sectioned into 4-μm-thick slices. Tissue sections were collected and stained with hematoxylin and eosin (H&E) (Servicebio, Wuhan, China) following the manufacturer’s protocol.

### 4.12. Immunohistochemistry IHC Staining

Paraffin-embedded sections were dewaxed in xylene and rehydrated through a graded alcohol series. Endogenous peroxidase activity was blocked by incubation with 3% H_2_O_2_ for 10 min at room temperature. Sections were then incubated overnight at 4 °C with the following primary antibodies (all from Abcam, UK): anti-myosin heavy chain/MYH3 (clone F1.652; 1:200 dilution), CD68 (clone E307V; 1:100), and CD206/MRC1 (clone E6T5J; 1:200). Subsequently, sections were incubated with biotin-conjugated secondary antibodies at 4 °C for 50 min. After three washes with PBS, streptavidin-conjugated horseradish peroxidase (HRP) was applied, followed by development with 3,3′-diaminobenzidine (DAB) substrate. Chromogenic reaction was monitored in real-time by microscopy. Sections were then rinsed with distilled water, counterstained with hematoxylin, differentiated in 1% acid ethanol for 1 s, rinsed in tap water, blued in 0.1% ammonia solution, and finally washed under running water. Dehydration was performed through a graded ethanol series (70%, 95%, 100%), followed by clearing in xylene and mounting with neutral gum. Three representative fields per sample were imaged for analysis.

### 4.13. Immunostaining and Image Analysis

The paraffin sections were fixed in 4% PFA, followed by counterstaining with the nuclear dye DAPI. For immunostaining, cells were blocked for 1–2 h in 5% goat serum before incubation with primary and secondary antibodies. The primary antibodies were: anti-Pax-7 antibody (clone EE-8; 1:50; Santa Cruz Biotechnology, Inc., Santa Cruz, CA, USA), anti-laminin 1:50, anti-fibronectin 1:50, anti-fast skeletal myosin heavy chain 1:200, and anti-slow skeletal myosin heavy chain 1:500 (Abcam, UK). Image J was used to count the number of cells.

### 4.14. Detection of Cytokines by ELISA

The serum was centrifuged at 1500× *g* for 15 min at 4 °C and the supernatant was collected. The levels of IL-6, IL-1β, TNF-α and IL-10 were obtained using the enzyme-linked immunoabsorbent assay (ELISA) kits (Nanjing Jiancheng Biocompany, Nanjing, China) in accordance with the manufacturer instructions.

### 4.15. Western Blot

Muscle proteins were extracted using a nondenaturing lysis buffer (e.g., Cell Signaling Technology, Danvers, MA, USA; Cat. # 9803) according to the manufacturer’s protocol. Protein concentrations were normalized using a BCA assay (Thermo Fisher Scientific (Waltham, MA, USA)), followed by biotinylation and antibody incubation. Cells for Western blot were grown for 3 h or 72 h in wells coated (treatment) in 6-well plates, and lysed in RIPA buffer (Sigma (St. Louis, MO, USA)). After adjustment of protein concentrations (as determined by BCA assays), samples were boiled in Laemmli buffer and used for Western blot. Antibodies used were: phospho-p44/42 MAPK (1:500, Abcam), p44/42MAPK (1:10,000, Abcam), phospho-p38 MAPK (1:800, Gene Tex), p38 MAPK (1:800, Gene Tex), phospho-FAK (1:1000, Abcam), FAK (1:2000, Abcam), Laminin (1:2000, Novus), β-actin (1:1000, Abcam), GAPDH (1:2500, Abcam). Image J was used to analyze the images.

### 4.16. Quantitative PCR and Analysis

RNA was extracted from frozen muscles with the RNeasy mini kit (Qiagen, Hilden, Germany) following manufacturer’s instructions. Typically, 500 ng of total RNA were subjected to reverse transcription (RT) with FastKing one-step method (Tiangen). RT-PCR was performed using TB Green Premix Ex Taq (TliRNaseH Plus) to evaluate expression. Primers used were FN1-F: GGCCACACCTACAACCAGTA, FN1-R: TCGTCTCTGTCAGCTTGCAC, mGapdh-F: GTCAAGGCCGAGAATGGGAA and mGapdh-R: CTCGTGGTTCACACCCATCA. The relative expression of targ3.17.et genes was measured by the DDCt method according to:

ΔΔCt = experimental group (Ct target gene − Ct reference gene) − control group (Ct target gene − Ct reference gene)

The fold-change in mRNA expression was calculated by the 2^−ΔΔCt^ method.

### 4.17. Flow Cytometric Analysis

Cell suspensions were obtained from triturated thymus and passed through a 200 µm nylon mesh. Flow cytometric analysis data were collected using the BD Fortessa Cell analyzer and analyzed utilizing the Kaluza software version 2.1 (Beckman Coulter, Brea, CA, USA). The following antibodies were purchased from BioLegend (San Diego, CA, USA): APC/Cyanine7 anti-mouse CD45 Antibody (103116), PE anti-mouse CD4 Antibody (100512), PerCP anti-mouse CD8a Antibody (100732), Alexa Fluor^®^ 488 anti-mouse CD3 antibody (100210), and APC anti-mouse CD25 antibody (101910).

### 4.18. Statistics

For in vivo studies, data were presented as means ± standard deviation (SD) and assessed for significance by a *t*-test, as appropriate. *p*-values were determined for multiple comparisons using the Bonferroni correction. Values of *p*  ≤  0.05 (*), *p*  ≤  0.01 (**), *p*  ≤  0.005 (***), and *p*  ≤  0.001 (****) were considered significant. Statistical tests were conducted using GraphPad Prism 8.0.

## Figures and Tables

**Table 1:** Inflammatory cytokine levels.

Cytokine	Group	Control (Mean ± SEM, *n*)	Treatment (Mean ± SEM, *n*)	Change	*p*-Value
IL-1β	rhFN-NM	46.839 ± 4.888 (*n* = 3)	35.886 ± 4.794 (*n* = 4)	↓	**0.0371** *
	mFN	46.839 ± 4.888 (*n* = 3)	42.315 ± 9.035 (*n* = 3)	↓	0.4918
IL-6	rhFN-NM	54.353 ± 3.299 (*n* = 3)	47.738 ± 7.337 (*n* = 4)	↓	0.2109
	mFN	54.353 ± 3.299 (*n* = 3)	45.459 ± 10.862 (*n* = 3)	↓	0.2654
TNF-α	rhFN-NM	349.998 ± 42.737 (*n* = 3)	291.676 ± 44.842 (*n* = 4)	↓	0.1229
	mFN	349.998 ± 42.737 (*n* = 3)	253.707 ± 95.844 (*n* = 3)	↓	0.1943
IL-10	rhFN-NM	488.6 ± 171.5 (*n* = 3)	597.7 ± 122.9 (*n* = 4)	↑	0.3642
	mFN	488.6 ± 171.5 (*n* = 3)	556.7 ± 210.9 (*n* = 3)	↑	0.6813

Notes: ↑: increase; ↓: decrease (vs. control); control: ntreated geriatric mice with CTX injury; *: significant (*p* < 0.05); bold highlights significance; ↑ (NS): value increased but not statistically significant (*p* > 0.05).

**Table 2:** Structures of integrin.

Region	Template	Sequence Identity	Method
Integrin-α7	4MMX	28.44%	Homology modeling
Integrin-β1	4MMX	45.26%	Homology modeling
Integrin-α5	4MMX	100.00%	Crystal structure
Integrin-β1	4MMX	45.26%	Homology modeling
Integrin-α5	4MMX	100.00%	Crystal structure
Integrin-β3	4MMX	100.00%	Crystal structure
Integrin-α4	4IRZ	100.00%	Crystal structure
Integrin-β1	4MMX	45.26%	Homology modeling

**Table 3:** Structures of fibronectin.

Region	Template	Sequence Identity	Method
1–47	NO	NO	De novo modeling
48–140	1o9a	100%	Crystal structure
93–182	2cg7	100%	Crystal structure
183–275	1fbr	100%	Crystal structure
276–297	NO	NO	De novo modeling
298–603	3m7p	100%	Crystal structure
604–803	2ha1	100%	Crystal structure
783–996	3t1w	27.63%	Homology modeling
903–1268	6mfa	99.73%	Crystal structure
1173–1539	3t1w	100%	Crystal structure
1270–1626	1fnf	82.07%	Homology modeling
1542–1887	3t1w	33.33%	Homology modeling
1632–1898	4gh7	31.84%	Homology modeling
1814–2081	1fnh	100%	Crystal structure
2081–2446	NO	NO	De novo modeling

## Data Availability

Data is contained within the article.
